# Is metabolic generalism the Breakfast of Champions for pathogenic *Candida* species?

**DOI:** 10.1371/journal.ppat.1012752

**Published:** 2024-12-11

**Authors:** Delma S. Childers, Jane Usher

**Affiliations:** 1 University of Aberdeen, Institute of Medical Sciences, Aberdeen Fungal Group, Aberdeen, United Kingdom; 2 Medical Research Council Centre for Medical Mycology, University of Exeter, Exeter, United Kingdom; Vallabhbhai Patel Chest Institute, INDIA

“Metabolic generalism” refers to the ability of an organism to utilise a wide range of nutrients and flexibly adapt to dynamic environmental conditions. For *Candida* spp., this versatility is crucial for their survival and virulence in diverse host niches [1], allowing them to thrive. In contrast, metabolic specialists follow a strict hierarchy of carbon source utilisation. While this hierarchy does not preclude specialists from potentially utilising a wide range of nutrients, it could delay switching to alternative carbon sources during transitions between host niches. In this Pearl, we will explore major metabolic themes supporting *Candida* species pathogenesis and highlight how the metabolic specialist, *Candida glabrata*, provides insights into what metabolic determinants really matter for survival in the host.

## 1: Common and uncommon metabolic themes in human fungal pathogens versus other fungi

A recent large-scale metabolic analysis of 853 fungal strains from diverse phylogenies suggests that only ~10% of yeasts are metabolic specialists [2]. Within that grouping, only *C*. *glabrata*, which has recently been renamed to *Nakaseomyces glabratus*, is a human pathogen. This places *C*. *glabrata* in a unique position that runs counterintuitive to the *Candida albicans* paradigm that dynamic host microenvironments require rapid pathogen adaptation facilitated by metabolic generalism [3]. Limited data are available on *C*. *glabrata* metabolic adaptation within the host, leaving much to be learned about the metabolic factors necessary for its pathogenesis. Interestingly, *Saccharomyces cerevisiae* and *C*. *albicans* were classified as “Normal” in this metabolic analysis, narrowly missing the specialist and generalist classifications we would classically assign to each species. We are considering *S*. *cerevisiae* and *C*. *albicans* as examples of metabolic specialists and generalists for the purposes of this article.

*Candida* species have evolved metabolic pathways and regulatory mechanisms to thrive in the human host environment, demonstrating remarkable resource range and adaptability. *C*. *albicans* is well-known for its metabolic flexibility and ability to metabolize a broad range of carbon sources including N-acetylglucosamine (GlcNAc), a monosaccharide derived from host glycoproteins and glycolipids. *C*. *glabrata* also exhibits significant adaptability to the host environment, albeit through different mechanisms due to its divergent evolutionary background. *C*. *glabrata* does not rely on GlcNAc as a primary carbon source but has instead developed alternative metabolic strategies to survive in nutrient-limited conditions. For instance, *C*. *glabrata* can efficiently utilise non-fermentable carbon sources such as glycerol, fatty acids, and amino acids, especially when glucose is scarce. This metabolic versatility allows *C*. *glabrata* to prosper in exigent host environments, such as the bloodstream, where glucose levels are high compared to most host niches (approximately 0.1%) [4], and low oxygen niches where other carbon sources are more prevalent. However, our knowledge around metabolic plasticity is limited to just a few strains per fungal species [5]. There is much better depth of understanding about intraspecies diversity in resource management and how this impacts colonisation and disease in bacterial pathogens, such as *Salmonella* [6].

Finally, metabolising or detoxifying host-derived compounds and the production of tissue-damaging enzymes is fundamental to *Candida* species’ capacity to cause disease [7]. Proteases, such as secreted aspartyl proteases or yapsins [8], can support nutrient sensing or regulation, nutrient acquisition and contribute to host immune evasion and damage. Metabolism also influences host immune evasion and damage by contributing precursors to cell wall biosynthesis and regulating production of other virulence factors, like EPA adhesins. Further, glutathione and trehalose production serve a critically protective role against oxidative stressors, like those imposed by innate immune cells. Both glutathione and trehalose are involved in resistance to antifungal agents, and trehalose can also be used as a carbon storage system [9]. *C*. *glabrata* excels at managing oxidative stress, a critical factor for survival in the hostile host immune system environment and can withstand higher H_2_O_2_ concentrations than even *C*. *albicans* [10].

## 2: Is metabolic generalism essential in vivo?

In *S*. *cerevisiae*, the presence of vanishingly low amounts of glucose (approximately 0.02%) [11] activates glucose repression and carbon catabolite inactivation pathways. Glucose repression results in alternative carbon transcript degradation and repression, while carbon catabolite inactivation triggers ubiquitination and proteasomal degradation of enzymes involved in the glyoxylate, gluconeogenic, and other alternative carbon source assimilation pathways.

**Fig 1:**
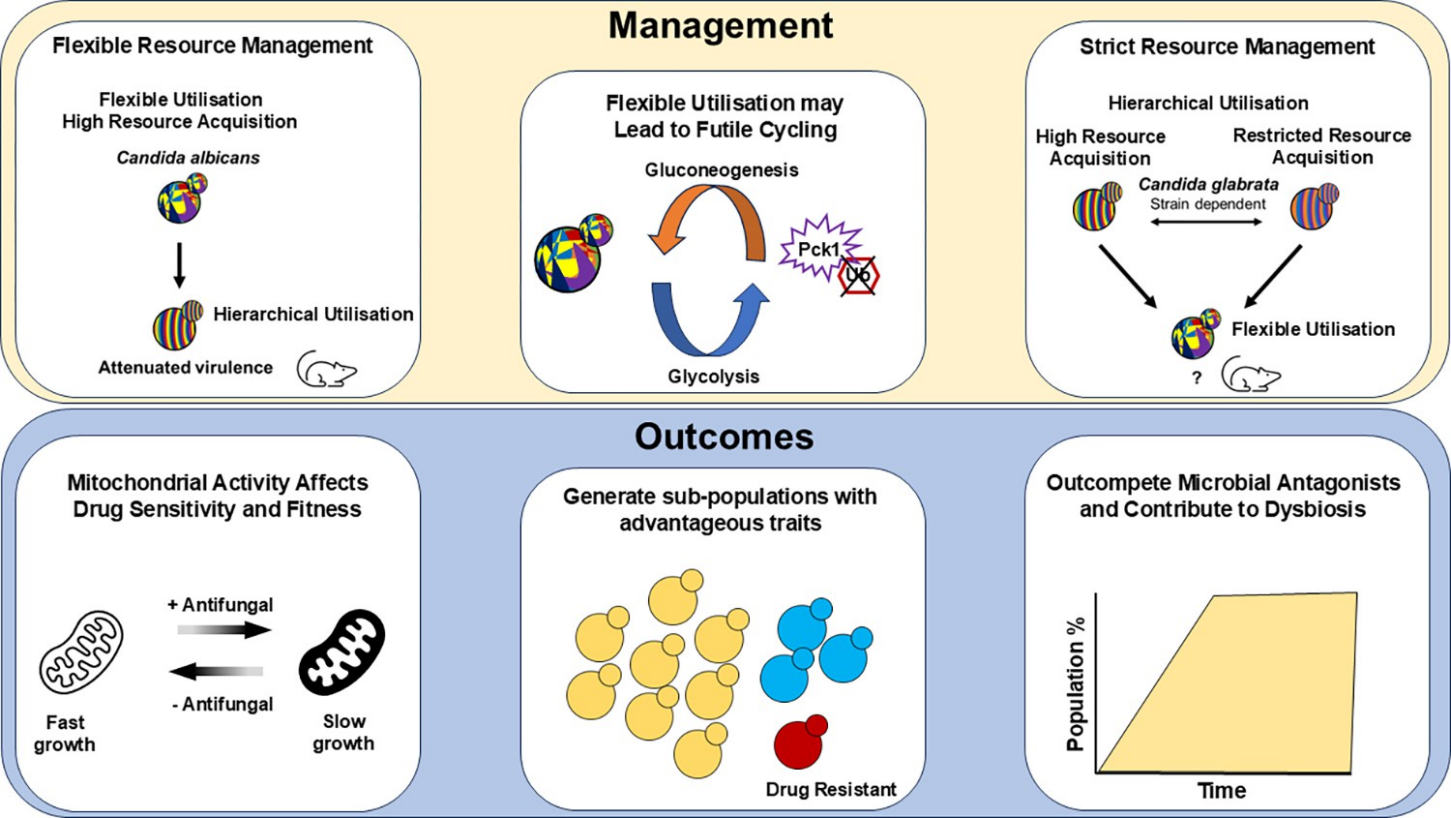
Resource management and metabolic strategies affect virulence attributes and disease outcomes. Most *Candida* species exhibit flexible resource management and metabolic generalism. Engineering specialist strains with a more hierarchical resource utilisation strategy results in attenuated virulence in *C*. *albicans*. However, the biochemical danger underlying metabolic flexibility is futile cycling. Futile cycling is energetically disadvantageous, but its significance to in vivo fitness is unknown. Strict resource management and metabolic specialism were disadvantageous to *S*. *cerevisiae* fitness in vivo, but its role in *C*. *glabrata* pathogenesis is still untested. These different metabolic strategies can affect mitochondrial activity, growth rate, and antifungal resistance of pathogens. This, in turn, can influence population heterogeneity and competitive fitness against microbial antagonists.

These responses are an essential part of what makes *Saccharomyces* species metabolic specialists, and *C*. *albicans*, despite being a metabolic generalist, has an intact glucose repression pathway [12]. However, *C*. *albicans* can quickly switch between different metabolic pathways due partly to evolutionary rewiring of key ubiquitination targets [3,13]. The glyoxylate cycle and gluconeogenic enzymes, Icl1 and Pck1, are insensitive to carbon catabolite inactivation due to lost ubiquitination motifs [13], allowing a short overlapping period where cells can have active alternative carbon assimilation simultaneously with glycolysis. *C*. *albicans* strains bearing *ICL1* with restored ubiquitination motifs were less fit when colonising the murine gastrointestinal tract and had significantly reduced virulence in systemic infection compared to strains with wild-type *ICL1* alleles. Thus, metabolic flexibility is essential for *C*. *albicans* systemic survival and gastrointestinal colonisation [3], further strengthening the argument that host niches impose dynamic nutritional restrictions on fungal pathogens (Fig 1). The Icl1 ubiquitination motif is also absent in *Candida tropicalis* and *Candida parapsilosis*, suggesting that metabolic flexibility conferred through restricted carbon catabolite inactivation is important more broadly in CUG-clade *Candida* species (recently taxonomically renamed to Serinales [14]). While we might assume that most *Candida* species fit the *C*. *albicans* metabolic model, emerging pathogenic species like *Candida auris* have likely independently evolved metabolic strategies for host fitness that require further study.

## 3: Does metabolic specialism support pathogenesis?

Understanding the molecular basis of metabolic specialism and regulation in human fungal pathogens is critical, particularly in the context of in vivo environments where these pathogens must adapt to survive and cause disease. *S*. *cerevisiae* is not normally a human pathogen but can cause infections in severely immunocompromised patients. Both metabolic specialist and generalist clinical isolates have been identified for *S*. *cerevisiae*, and direct comparisons of these non-isogenic strains suggest that metabolic generalists have better intracellular survival in macrophages than specialist isolates [3]. Further, isogenic *S*. *cerevisiae* strains engineered to be generalists by inactivating the glucose-induced degradation complex were more fit and virulent in an immune-compromised murine systemic infection model [3]. Altogether, these data suggested that metabolic specialism can cause disease in certain contexts, but generalism appears to be more fit in vivo. While *C*. *glabrata* is a metabolic specialist that can be highly fermentative in vitro, it exhibits some metabolic flexibility by efficiently utilizing alternative carbon sources such as glycerol, lactate, and amino acids, which are prevalent in distinct host niches. Genes involved in the utilisation of non-glucose carbon sources, such as glycerol-3-phosphate dehydrogenase (*GPD1*) and *ICL1*, are up-regulated during infection, indicating a shift in metabolic strategy to adapt to the host environment. In addition, *C*. *glabrata* can utilise glycerol as a carbon source in vivo under osmotic stress conditions. The up-regulation of genes such as *GPD2* (glycerol-3-phosphate dehydrogenase) in response to osmotic stress and the ability to produce trehalose underscores its adaptability to fluctuating environmental conditions within host tissues.

Further, while *C*. *glabrata* Icl1 has a conserved glucose-induced ubiquitination motif, Pck1 does not have a high confidence ubiquitination motif [3]. These in silico data suggest that gluconeogenesis could be active when *C*. *glabrata* cells transition to glucose-containing environments, like the bloodstream. Maintaining gluconeogenic activity during active glycolysis in the presence of glucose would plunge cells into an energetically unfavourable futile cycle. Futile cycling in *S*. *cerevisiae* strains engineered to express gluconeogenic enzymes during glycolysis [15] was not fatal and only had modest effects on growth rate. These findings pose several questions: Does futile cycling help provide intermediate metabolic products for stress adaptation or virulence responses? Is metabolic flexibility via futile cycling necessary for *C*. *glabrata* survival in the host? And if futile cycling is not disadvantageous, why do metabolic specialists maintain a strict hierarchy of resource acquisition? Further work is needed to address these questions.

## 4: Is mitochondrial function required in vivo?

In *C*. *glabrata*, the role of mitochondria and respiratory metabolism is increasingly recognised as vital for survival and virulence in the host environment. Disruption of mitochondrial respiratory function, typically through loss of mitochondrial DNA (mtDNA) in *C*. *glabrata*, results in impaired lipid homeostasis, growth, and reduced virulence, which highlights the importance of mitochondria in adapting to the host environment. mtDNA mutations can lead to defects in the respiratory chain, affecting the production of ATP and ROS. Resistance to azole antifungal drugs often involves mutations that affect mitochondrial function [16]. This resistance can be indirectly attributed to the role of mitochondria in synthesising the heme used by cytochrome P450 enzymes in ergosterol biosynthesis, a pathway targeted by azoles [17]. Mitochondria also influence the expression of drug efflux pumps, which are critical for azole resistance and are strongly up-regulated in petite and mitochondrial mutant cells. This connection underlines the importance of mitochondrial health for the maintenance of drug resistance mechanisms in *C*. *glabrata*. Further, the *C*. *glabrata* mitochondrial genome is highly diverse between isolates, which has implications for its role in antifungal drug resistance, host interactions, and suggests this organelle may be undergoing rapid evolution [18].

In *C*. *glabrata*, mitochondria are important for energy production, particularly in the form of ATP, through oxidative phosphorylation. Unlike *C*. *albicans*, which can undergo significant metabolic shifts to fermentation in vivo, *C*. *glabrata* relies more on mitochondrial respiration, especially in environments where glucose is limited [19]. This reliance suggests that mitochondrial function is critical for its energy metabolism and overall survival in vivo. Despite the fundamental role that mitochondria play in metabolism, petites or small colony variants that result from mitochondrial loss have been isolated from human bloodstream infections [20]. Petite mutants are generally less fit than wild-type cells in vitro, but can be hypervirulent in murine infection models and show high resistance and tolerance [21] to azoles and echinocandins. Thus, mitochondrial activity could be expendable in selective conditions, such as antifungal insults.

## 5: Is rapid proliferation the key to success?

For opportunistic pathogens, virulence is tightly linked with changes in host status. Rapid proliferation and increasing pathogen density can thus be key virulence determinants in compromised hosts [22]. A recent clinical study linked hematopoietic cell transplant mortality to expansions in *C*. *parapsilosis* complex species density, leading to fungal dysbiosis and likely contributing to poor patient outcomes [23]. Targeting mechanisms involved in niche expansion and proliferation could be beneficial to combatting fungal dysbiosis and disease.

*S*. *cerevisiae* is a useful example of the principle that metabolism supporting rapid proliferation can give an organism a competitive advantage. By rapidly consuming available glucose and turning this into ethanol, *S*. *cerevisiae* creates a hostile environment for competing bacteria while enabling its own rapid proliferation and securing its niche. Similar strategies may be employed by fungal pathogens. The *C*. *albicans eed1Δ/Δ* mutant lacks a quintessential virulence factor, hyphal formation, but is still as virulent as wild-type cells in vivo [24]. *eed1Δ/Δ* also reached higher in vivo fungal burdens than the filamentation-competent wild-type strain and had increased in vitro fitness on alternative carbon sources. Altogether, these data indicate that metabolic flexibility and rapid proliferation can be just as important for virulence as hyphal formation.

One condition where rapid proliferation is detrimental is during antifungal treatment. Slow growth promotes antifungal tolerance and resistance against multiple drug classes. Drug-resistant strains are often less fit than susceptible strains in the absence of drug, though there is growing evidence for compensatory mutations that allow drug resistance without fitness disadvantages [25]. Therefore, the winning metabolic strategy is likely a balance between regulatory programmes that allow rapid division under permissive conditions while maintaining a diverse subpopulation of metabolically less active cells that can ride out the wave of antifungal counterattack, ensuring genetic survival.

## 6: Perspectives and conclusion

Metabolic flexibility provides *Candida* species with the necessary tools to adapt, survive, and thrive in the diverse and challenging environments within the human host. *C*. *glabrata* is the only metabolic specialist yeast known to survive at 37°C and cause disease. As new pathogens emerge, other specialists may be identified with diverse metabolic programmes. It is, therefore, vital to understand how metabolism primes these pathogens for success and use this knowledge to inform novel interventions and diagnostics.

## References

[1] Mayer FL, Wilson D, Hube B. *Candida albicans* pathogenicity mechanisms. Virulence. 2013 Feb 15;4(2):119–28. doi: 10.4161/viru.22913 Epub 2013 Jan 9. ; PMCID: PMC3654610. 23302789 PMC3654610

[2] Opulente DA, LaBella AL, Harrison MC, Wolters JF, Liu C, Li Y, et al. Genomic factors shape carbon and nitrogen metabolic niche breadth across *Saccharomycotina* yeasts. Science (80-). 2024;384(6694). 10.1126/science.adj4503 PMC11298794 38662846

[3] Childers DS, Raziunaite I, Mol Avelar G, Mackie J, Budge S, Stead D, et al. The Rewiring of Ubiquitination Targets in a Pathogenic Yeast Promotes Metabolic Flexibility, Host Colonization and Virulence. PLoS Pathog. 2016;12(4):1–26. 10.1371/journal.ppat.1005566 PMC4830568 27073846

[4] Barelle CJ, Priest CL, Maccallum DM, Gow NA, Odds FC, Brown AJ. Niche-specific regulation of central metabolic pathways in a fungal pathogen. Cell Microbiol. 2006 Jun;8(6):961–71. doi: 10.1111/j.1462-5822.2005.00676.x ; PMCID: PMC1472618. 16681837 PMC1472618

[5] Usher J, Ribeiro GF, Childers DS. The *Candida glabrata* Parent Strain Trap: How Phenotypic Diversity Affects Metabolic Fitness and Host Interactions. Microbiol Spectr. 2023;11(1). 10.1128/spectrum.03724-22 PMC9927409 36633405

[6] Seif Y, Kavvas E, Lachance JC, Yurkovich JT, Nuccio SP, Fang X, et al. Genome-scale metabolic reconstructions of multiple *Salmonella* strains reveal serovar-specific metabolic traits. Nat Commun. 2018;9:3771. doi: 10.1038/s41467-018-06112-5 30218022 PMC6138749

[7] Weerasinghe H, Stölting H, Rose AJ, Traven A, Weerasinghe H, Stölting H, et al. *Candida glabrata*. Nat Commun. 2024;13(1):1–20. doi: 10.1038/s41467-021-24095-8

[8] Askari F, Rasheed M, Kaur R. The yapsin family of aspartyl proteases regulate glucose homeostasis in *Candida glabrata*. J Biol Chem. 2022;298(2):101593. doi: 10.1016/j.jbc.2022.101593 35051415 PMC8844688

[9] Van Ende M, Timmermans B, Vanreppelen G, Siscar-Lewin S, Fischer D, Wijnants S, et al. The involvement of the *Candida glabrata* trehalase enzymes in stress resistance and gut colonization. Virulence. 2021;12(1):329–45. doi: 10.1080/21505594.2020.1868825 33356857 PMC7808424

[10] Cuéllar-Cruz M, Briones-Martin-del-Campo M, Cañas-Villamar I, Montalvo-Arredondo J, Riego-Ruiz L, Castaño I, et al. High resistance to oxidative stress in the fungal pathogen *Candida glabrata* is mediated by a single catalase, Cta1p, and is controlled by the transcription factors Yap1p, Skn7p, Msn2p, and Msn4p. Eukaryot Cell. 2008;7(5):814–25. 18375620 10.1128/EC.00011-08 PMC2394966

[11] Meijer MMC, Boonstra J, Verkleij AJ, Verrips CT. Glucose repression in *Saccharomyces cerevisiae* is related to the glucose concentration rather than the glucose flux. J Biol Chem. 1998;273(37):24102–7. doi: 10.1074/jbc.273.37.24102 9727030

[12] Rodaki A, Bohovych IM, Enjalbert B, Young T, Odds FC, Gow NA, et al. Glucose promotes stress resistance in the fungal pathogen *Candida albicans*. Mol Biol Cell. 2009;20(22):4845–55. Epub 2009/09/18. E09-01-0002 [pii] doi: 10.1091/mbc.E09-01-0002 ; PubMed Central PMCID: PMC2777113. 19759180 PMC2777113

[13] Sandai D, Yin Z, Selway L, Stead D, Walker J, Leach MD, et al. The evolutionary rewiring of ubiquitination targets has reprogrammed the regulation of carbon assimilation in the pathogenic yeast *Candida albicans*. MBio. 2012;3(6). 10.1128/mBio.00495-12 PMC3520108 23232717

[14] Groenewald M, Hittinger CT, Bensch K, Opulente DA, Shen XX, Li Y, et al. A genome-informed higher rank classification of the biotechnologically important fungal subphylum *Saccharomycotina*. Stud Mycol. 2023 Jun;105:1–22. doi: 10.3114/sim.2023.105.01 ; PMCID: PMC11182611. 38895705 PMC11182611

[15] Navas MA, Cerdan S, Gancedo JM. Futile cycles in *Saccharomyces cerevisiae* strains expressing the gluconeogenic enzymes during growth on glucose. Proc Natl Acad Sci U S A. 1993;90(4):1290–4. 8381962 10.1073/pnas.90.4.1290 PMC45858

[16] Shingu-Vazquez M, Traven A. Mitochondria and fungal pathogenesis: Drug tolerance, virulence, and potential for antifungal therapy. Eukaryot Cell. 2011;10(11):1376–83. doi: 10.1128/EC.05184-11 21926328 PMC3209048

[17] Crešnar B, Petrič S. Cytochrome P450 enzymes in the fungal kingdom. Biochim Biophys Acta. 2011 Jan;1814(1):29–35. doi: 10.1016/j.bbapap.2010.06.020 Epub 2010 Jul 7. . 20619366

[18] Helmstetter N, Chybowska AD, Delaney C, Da Silva Dantas A, Gifford H, Wacker T, et al. Population genetics and microevolution of clinical *Candida glabrata* reveals recombinant sequence types and hyper-variation within mitochondrial genomes, virulence genes, and drug targets. Genetics. 2022;221(1). 10.1093/genetics/iyac031 PMC9071574 35199143

[19] Ene IV, Brunke S, Brown AJP, Hube B. Metabolism in fungal pathogenesis. Cold Spring Harb Perspect Med. 2014;4(12):1–21. doi: 10.1101/cshperspect.a019695 25190251 PMC4292087

[20] Badrane H, Cheng S, Dupont CL, Hao B, Driscoll E, Morder K, et al. Genotypic diversity and unrecognized antifungal resistance among populations of *Candida glabrata* from positive blood cultures. Nat Commun. 2023;14(1). 10.1038/s41467-023-41509-x PMC10516878 37739935

[21] Torres EM, Sokolsky T, Tucker CM, Chan LY, Boselli M, Dunham MJ, et al. *Candida albicans* and *Candida glabrata* clinical isolates exhibiting reduced echinocandin susceptibility. Antimicrob Agents Chemother. 2013 Apr 23;8(5):2892–4. doi: 10.1007/s11908-016-0554-5 PMC1538661 16870797

[22] Wollein Waldetoft K, Råberg L, Lood R. Proliferation and benevolence—A framework for dissecting the mechanisms of microbial virulence and health promotion. Evol Appl. 2020;13(5):879–88. doi: 10.1111/eva.12952 32431740 PMC7232753

[23] Yu C, Bonaduce MJ, Klar AJS. Remarkably high rate of DNA amplification promoted by the mating-type switching mechanism in *Schizosaccharomyces pombe*. Genetics. 2012 May;191(1):285–9. 22377633 10.1534/genetics.112.138727 PMC3338267

[24] Ganguly S, Bishop AC, Xu W, Ghosh S, Nickerson KW, Lanni F, et al. Zap1 control of cell-cell signaling in *Candida albicans* biofilms. Eukaryot Cell. 2011;10(11):1448–54. 21890817 10.1128/EC.05196-11 PMC3209068

[25] Cowen LE, Steinbach WJ. Stress, drugs, and evolution: The role of cellular signaling in fungal drug resistance. Eukaryot Cell. 2008;7(5):747–64. doi: 10.1128/EC.00041-08 18375617 PMC2394973

